# Bullying, Psychological, and Physical Trauma During Early Life Increase Risk of Major Depressive Disorder in Adulthood: A Nationwide Community Sample of Korean Adults

**DOI:** 10.3389/fpsyt.2022.792734

**Published:** 2022-03-25

**Authors:** Hyun Soo Kim, Hong Jin Pyo, Maurizio Fava, David Mischoulon, Mi Jin Park, Hong Jin Jeon

**Affiliations:** ^1^Department of Psychiatry, Depression Center, Samsung Medical Center, Sungkyunkwan University School of Medicine, Seoul, South Korea; ^2^Depression Clinical and Research Program, Massachusetts General Hospital, Harvard Medical School, Boston, MA, United States; ^3^Department of Health Sciences and Technology, Department of Medical Device Management and Research, and Department of Clinical Research Design and Evaluation, Samsung Advanced Institute for Health Sciences and Technology (SAIHST), Sungkyunkwan University, Seoul, South Korea

**Keywords:** early life trauma, bullying, emotional neglect, psychological trauma, major depressive disorder

## Abstract

**Background:**

There is an association between early life traumas and the development of depression in adults. Few studies have used nationwide population-based samples to investigate whether the type of early life trauma differentially influences the risk of developing depression.

**Methods:**

Major depressive disorder and early life trauma were assessed using the Korean version of the Composite International Diagnostic Interview (K-CIDI) for DSM-IV psychiatric disorder and a questionnaire for early life trauma in the Korean Epidemiological Catchment Area Study in 2016. A total of 4,652 participants were included in the final analysis. This study evaluated the effect of the type and frequency of reported early life trauma on the risk of developing MDD and the association between reported early life trauma and differential symptoms of MDD.

**Results:**

Individuals with reported early life trauma had a 3.7-fold increased risk of MDD. The risk of MDD was associated with bullying trauma (odds ratio (OR) = 1.847, *p* = 0.005) after adjusting for age, gender, marriage, job, and education years. The risk of MDD was increased as the types of reported early life traumas increased.

**Conclusion:**

Bullying trauma during early life represents a risk factor for MDD, especially in individuals exposed to multiple traumas in early life.

## Introduction

Exposure to trauma in early life is associated with various psychopathologies and poor outcomes in adulthood (1). According to a World Health Organization (WHO) survey, early life trauma was highly prevalent and strongly associated with mental health disorders (2). Early life trauma can have a significant impact on biological, social, emotional, and cognitive functioning, with long-term consequences lasting into adulthood (3–5). Previous study reported that early life trauma was associated with poor mental health including depression and suicidality (6). A study of women in Turkey reported that dissociative disorder was correlated with the type and number of early life trauma (7).

Major depressive disorder (MDD) is one of the most prevalent psychiatric disorders, with a lifetime prevalence between 8% and 12% (8). The onset of MDD is associated with a broad range of demographic, clinical, and psychosocial variables (9). Early life trauma was shown to be an important risk factor for the development of MDD (10, 11). Early life trauma can alter the structure and function of the brain, and this has been posited as a potential link between this type of trauma and depression (5). In addition, alterations in the hypothalamic-pituitary-adrenal axis, such as glucocorticoid receptor sensitivity and increased immune reactivity, have been associated with both early life trauma and depression (12). From a clinical point of view, people with depression who have been maltreated or experienced early life trauma have an increased risk of resistance to treatment for depression (11).

Previous studies showed that early life trauma increases the risk of depression. However, it is inappropriate to regard early life trauma as a single phenomenon, so each type needs to be considered separately (13). To the best of our knowledge, few previous studies have examined the relationship between specific types of early life trauma and MDD in adulthood in a large nationwide general population sample (14). Moreover, most previous studies have focused on a single form of early life trauma without comparison to other traumas (15). Recently, study in early life trauma has expanded not only to the study of the effects of early life trauma on adult health, but also to the prevention of early life trauma (16). Therefore, understanding which types of early life trauma have a significant impact on the pathology of depression will help devise strategies for early trauma prevention. In addition, since trauma is a subjective experience, reaction to traumatic experiences may vary depending on religious and cultural backgrounds (17). It is necessary to compare the relationship between depression and early life trauma in a nationwide community sample of Koreans with previous studies in other cultural countries.

Thus, we attempted to characterize the relationship between different types of early life trauma and MDD using a nationwide community sample of South Koreans. The primary aim of the study was to identify the influence of the type and frequency of early life trauma on the presence of MDD. The secondary aim of the study was to explore the associations between early life trauma and the different symptoms of MDD.

## Methods

### Data Collection, Participants, and Interviewers

Data were collected by the 2016 Korean Epidemiological Catchment Area Study (KECA-2016) from April to November of 2016. The purpose of this study was to determine the lifetime and 12-month prevalence of mental disorders, socioeconomic correlates, and comorbidities every 5 years among Korean adults. The subjects of this study were ordinary adults over the age of 18 who live in the community. This study was intended to determine the prevalence of the general population and did not include people in hospitals or nursing homes. The respondents were evenly selected from 21 community catchment areas throughout the country by a stratified multistage cluster sampling method to generate national representative data. Since the population of each survey area was different, the number of subjects in each survey area was estimated by considering the ratio of the population of each survey area to the total population of Korea. Using the last-birthday method, the individual with the earliest birthday was randomly selected and interviewed. The institutional review board of Seoul National University College of Medicine approved all study procedures. All subjects were fully explained about the purpose and method of the study, and informed consent was obtained prior to participation. A total of 5,102 subjects aged over 18 years participated in face-to-face interviews.

The 147 interviewers with academic backgrounds in medicine, nursing, and social welfare living in the survey area were recruited from each catchment. The training of the interviewers was conducted by professional instructors in a central training center accredited by the WHO. The training was conducted for five full-time days by an instructor with qualifications of a psychiatrist or higher in accordance with the WHO recommendation standards. In addition to training for each CIDI session, mock interviews were conducted, and at the end, live interview with real patients were conducted. Live interviews with psychiatric patients followed by group discussions were conducted to confirm inter-rater reliability. For the qualitative management of the investigation, in addition to the interviewers, a field manager was assigned to supervise the interviewers, check the results of the investigation, and supervise the progress of the investigation.

### Measures

#### Korean Version of the Composite International Diagnostic Interview

The Korean version of the Composite International Diagnostic Interview (K-CIDI) version 2.1 was used for assessing participant mental health (18). The CIDI is a standardized diagnostic protocol to determine psychiatric diagnoses based on the Diagnostic and Statistical Manual of Mental Disorders, fourth edition (19). The K-CIDI is a translated version of the CIDI and was shown to be valid and reliable in a population aged from 18 to 65 years (19, 20). CIDI made according to the DSM IV diagnostic criteria was used, and therefore all psychiatric disorders diagnosed according to the DSM IV criteria. Section D of the K-CIDI on depression was used in this study. To identify early life trauma, Appendix A of the K-CIDI consisting of early life trauma questions was used. The inter-rater reliability, test/retest reliability, and validity of the K-CIDI are characterized by kappa values of 0.86–1.00, 0.42–0.89, and 0.50–1.00, respectively (18).

#### Early Life Trauma

The self-report questionnaire assessing early life trauma consisted of five items including bullying, emotional neglect, and psychological, physical, and sexual trauma. Bullying was evaluated using an item in the adverse childhood experiences international questionnaire (ACE-IQ) (21). Bullying was defined as “Bullying is when a young person or group of young people say or do bad and unpleasant things to another young person. It is also bullying when a young person is teased a lot in an unpleasant way or when a young person is left out of things on purpose. It is not bullying when two young people of about the same strength or power argue or fight or when teasing is done in a friendly and fun way.”

Emotional neglect, psychological trauma, physical trauma and sexual trauma was assessed using a questionnaire based on the NEMESIS-1 trauma questionnaire (22). Emotional neglect trauma was defined as “people at home didn't listen to you, your problems were ignored and/or you felt unable to find any attention or support from the people in your house.” Psychological trauma was defined as “you were cursed at, unjustly punished, your brothers and sisters were favored—but no bodily harm was done.” Physical trauma was defined as “being kicked, hit with or without an object, or being physically maltreated in any other way.” Sexual trauma was defined as “being touched sexually by anyone against your will, or being forced to touch anyone sexually, or pressured into sexual contact against your will.”

The experience of bullying trauma was measured by asking “How many times have you been bullied as you grew up, at or before the age of 18?” The experience of emotional neglect, psychological, physical, and sexual trauma was measured by asking “Growing up, do you think there was any kind of emotional neglect or psychological, physical, and sexual trauma at or before the age of 18?” The trauma questionnaire responses were coded on a 4-point scale (1: none, 2: once, 3: a few times, 4: many times). Based on the number of coding results, for each specific early life trauma, 1(none) was defined as those who did not experience early life trauma and 2 (once), 3 (a few times), 4 (many times) were defined as those who experienced early life trauma.

#### Major Depressive Disorder

The CIDI questionnaire for evaluating MDD was composed of DSM-IV criteria, and MDD was diagnosed according to the DSM-IV diagnostic criteria based on the evaluation results. In this study, among all participants, those diagnosed with MDD were classified as the MDD group, and those not diagnosed with MDD were classified as the control group.

### Statistical Analyses

For continuous variables, age and education year, the mean and standard deviation were presented, and the depression group and the control group were compared through an independent *t*-test. The categorical variables, such as gender, married status, occupation, type of trauma, and frequency of trauma were presented as numbers and percentages, and the depression group and the control group were compared through chi-square test.

In order to analyze the risk of developing MDD for early life trauma from various aspects, various types of trauma variables were created. To evaluate the risk of developing MDD for types of early life trauma, reported trauma was classified into five categories: bullying, emotional neglect, psychological, physical, and sexual trauma. In addition, in order to evaluate the overall impact of early life trauma, the group that reported even one trauma was classified into ‘any trauma'. Frequency of early life trauma was classified from ‘no trauma' to ‘5 trauma' according to the number of reported types of bullying, emotional neglect, psychological, physical, and sexual trauma.

For each type and frequency of reported early trauma variables, multivariable logistic regression analysis was performed including all explanatory variables within one model. The risk of developing MDD in adulthood was regressed onto each variables of reported early life trauma adjusted with age, gender, years of education, married status and occupation. For each multivariable logistic regression analysis, Bonferroni correction was conducted to adjust for multiple testing and statistical significance was set at *p* < 0.01 (0.05/5 = 0.01).

A chi-squared test was conducted to examine the association between reported early life trauma and the differential symptoms of MDD. Bonferroni correction was conducted to adjust for multiple testing and significance was set at *p* < 0.006 (0.05/9 = 0.006). Statistical analyses were conducted using the Statistical Package for Social Sciences version 17 (SPSS ver. 17).

## Results

### Demographics

The sociodemographic characteristics of the study population are presented in Table 1. A total of 5,102 participants were involved in the first study. However, 450 participants were excluded due to missing data and coding errors, leaving a total of 4,652 participants for the final analysis. Among the 4,652 participants, 216 (4.64%) were classified as MDD group and 4,436 (95.36%) were classified as control group.

**Table 1:** Comparison of the sociodemographic profiles of individuals with and without major depressive disorder (MDD) in participants from the Korean Epidemiological Catchment Area Study in 2016 (KECA-2016) (*N* = 4652).

	**Total** **(*n* = 4652)**	**MDD** **(*n* = 216)**	**Controls** **(*n* = 4436)**	**Statistics**
**Variables**	**No**.	**No**.	**No**.	***p*-value**
Mean age (SD)	49.78 (17.767)	47.18 (17.88)	49.91 (17.75)	0.027
Mean education year (SD)	12.14 (4.413)	12.00 (3.99)	12.15 (4.43)	0.636
Gender (%)				<0.001
- Female	2,851 (61.3%)	165 (76.4%)	2,686 (60.6%)	
- Male	1,801 (38.7%)	51 (23.6%)	1,750 (39.4%)	
Married status (%)				<0.001
- Married	2,846 (61.2%)	105 (48.6%)	2,741 (61.8%)	
- Widowed/separated/divorced	823 (17.7%)	49 (22.7%)	774 (17.4%)	
- Single	983 (21.1%)	62 (28.7%)	921 (20.8%)	
Occupation (%)				<0.001
- Full time	1,650 (35.5%)	45 (20.8%)	1,605 (36.2%)	
- Part time	443 (9.5%)	25 (11.6%)	418 (9.4%)	
- Unemployed	854 (18.4%)	56 (25.9%)	798 (18.0%)	
- Not indicated	1,705 (36.7%)	90 (41.7%)	1,615 (36.4%)	
Type of trauma (%)				
- Bullying trauma	382 (8.2%)	51 (23.6%)	331 (7.5%)	<0.001
- Emotional neglect trauma	440 (9.5%)	59 (27.3%)	381 (8.6%)	<0.001
- Psychological trauma	391 (8.4%)	59 (27.3%)	332 (7.5%)	<0.001
- Physical trauma	430 (9.2%)	54 (25.0%)	376 (8.5%)	<0.001
- Sexual trauma	165 (3.5%)	23 (10.6%)	142 (3.2%)	<0.001
Frequency of trauma (%)				<0.001
- No	3,729 (80.2%)	117 (54.2%)	3,612 (81.4%)	
- Single trauma	432 (9.3%)	31 (14.4%)	401 (9.0%)	
- Multiple traumas	491 (10.5%)	68 (31.5%)	423 (9.5%)	

*MDD, major depressive disorder; SD, standard deviation*.

*Independent t-test, chi-squared test*.

### Reported Early Life Trauma Frequency

Of the 216 individuals with MDD, 23.6 experienced bullying trauma, 27.3 experienced emotional neglect trauma, 27.3 experienced psychological trauma, 25 experienced physical trauma, and 10.6% experienced sexual trauma. Of the 4,36 individuals without MDD classified in the control group, 7.5 experienced bullying trauma, 8.6 experienced emotional neglect trauma, 7.5 experienced psychological trauma, 8.5 experienced physical trauma, and 3.2% experienced sexual trauma. The difference in reported trauma frequency between the depression group and the control group was statistically significant for all types of reported early life trauma.

### MDD and Type of Reported Early Life Trauma

The association between the type of reported early life trauma and the risk of developing MDD in adulthood is presented in Table 2. Overall, the risk of developing MDD increased 3.7 times for individuals who reported at least one early life trauma. Specifically, bullying trauma was associated with the risk of developing MDD (OR = 1.847, *p* = 0.005). Although not statistically significant, psychological trauma showed a marginal association with the risk of developing MDD (OR = 1.885, *p* = 0.011). For physical trauma, the original *p*-value was statistically significant, but it was not significant after Bonferroni correction (OR = 1.666, *p* = 0.023). Emotional neglect and sexual trauma were not associated with the risk of developing MDD.

**Table 2:** Results of multivariable logistic regression analysis of risk of developing major depressive disorder for specific types of reported early life trauma in participants from the Korean Epidemiological Catchment Area Study in 2016 (KECA-2016) (*n* = 4652).

	**Total sample (*****n*** **=** **4652)**		
	**MDD**		
**Early life trauma**	**AOR**	**95% CI**	***p*-value**
No trauma			
Any trauma	3.693^*^	2.771–4.921	<0.0001
No trauma			
Bullying trauma	1.847^*^	1.207–2.825	0.005
Emotional neglect trauma	1.537	0.975–2.422	0.064
Psychological trauma	1.885	1.154–3.080	0.011
Physical trauma	1.666	1.072–2.589	0.023
Sexual trauma	1.416	0.830–2.417	0.202

*MDD, major depressive disorder; AOR, adjusted odds ratio; CI, confidence interval*.

*Adjusted for age, gender, marriage, job, and education years*.

* *p < 0.01 in accordance with Bonferroni correction (p < 0.05 divided by 5)*.

### MDD and Frequency of Reported Early Life Trauma

The association between the frequency of reported early life trauma and the risk of developing MDD is presented in Table 3. The risk of developing MDD was increased as the types of reported early life traumas increased. Specifically, a significant OR, ranging from 1.6–3.7 for a single trauma, 2.3–5.8 for two types of trauma, 2.5–7.2 for three types of trauma, and 2.0–9.0 for four types of trauma, were associated with risk of MDD. Individuals who reported all five types of early life trauma had a 26-fold increased risk of MDD compared to individuals who reported none of the early life trauma. However, the estimate may be uncertain because of the wide confidence interval of 12.1–55.7.

**Table 3:** Results of multivariable logistic regression analysis of risk of developing major depressive disorder for frequency of reported early life trauma in participants from the Korean Epidemiological Catchment Area Study in 2016 (KECA-2016) (*n* = 4652).

	**Total sample (*****n*** **=** **4652)**		
	**MDD**		
**Early life trauma**	**AOR**	**95% CI**	***p*-value**
No trauma			
1 Trauma	2.480^*^	1.634–3.762	<0.0001
2 Traumas	3.695^*^	2.345–5.824	<0.0001
3 Traumas	4.263^*^	2.518–7.216	<0.0001
4 Traumas	4.289^*^	2.043–9.003	<0.0001
5 Traumas	26.033^*^	12.152–55.771	<0.0001

*MDD, major depressive disorder; AOR, adjusted odds ratio; CI, confidence interval*.

*Adjusted for age, gender, marriage, job, and education years*.

* *p < 0.01 in accordance with Bonferroni correction (p < 0.05 divided by 5)*.

### Differential Symptoms of MDD and Reported Early Life Trauma

The association between the different symptoms of MDD and reported early life trauma is presented in Table 4. No significant statistical values were found in all areas of the differential symptoms of MDD. Although not statistically significant, among the differential symptoms, depressed mood showed a marginal association with reported early life trauma (*p* = 0.008).

**Table 4:** Comparison of the differential symptoms of major depressive disorder according to the presence or absence of early life trauma in participants reported as major depressive disorder in the Korean Epidemiological Catchment area study in 2016 (KECA-2016) (*n* = 216).

	**MDD (*****n*** **=** **216)**		
	**With early life trauma (*n* = 99)**	**Without early life trauma (*n* = 117)**	**Statistics**
**Differential symptoms of MDD**	**No. (%)**	**No. (%)**	***p*-value**
Depressed mood	86 (86.9%)	113 (96.6%)	0.008
Diminished interest or pleasure	87 (87.9%)	96 (82.1%)	0.236
Fatigue or loss of energy	93 (93.9%)	111 (94.9%)	0.766
Weight loss or gain, appetite decrease or increase	88 (88.9%)	105 (89.7%)	0.839
Insomnia or hypersomnia	93 (93.9%)	103 (88.0%)	0.136
Psychomotor agitation or retardation	78 (78.8%)	77 (65.8%)	0.035
Worthlessness or guilty feeling	74 (74.7%)	81 (69.2%)	0.370
Diminished ability to think or concentrate	92 (92.9%)	106 (90.6%)	0.537
Thoughts of death, suicidal ideation, attempt, or plan	58 (58.6%)	78 (66.7%)	0.220

*MDD, major depressive disorder*.

*Chi-squared test*.

*p < 0.006 in accordance with Bonferroni correction (p < 0.05 divided by 9, the number of variables)*.

## Discussion

We attempted to characterize the relationship between reported early life trauma and MDD using a nationwide community sample of South Koreans. The aim was to identify the effect of reported early life trauma on the presence of MDD and explore the associations between reported early life trauma and the different symptoms of MDD. The bullying trauma during early life was risk factors for developing MDD in adulthood. Although not statistically significant, psychological trauma showed a marginal association with the risk of developing MDD. The risk of developing MDD was increased as the types of reported early life traumas increased. In participants with MDD, although not statistically significant, depressed mood showed a marginal association with reported early life trauma.

Among early life traumas, bullying trauma was the only risk factor for developing MDD in adulthood. These results were meaningful in that there are few studies comparing bullying trauma with other early life traumas. In addition, these results suggested that not only trauma in the family, but also trauma in school or peer relationships had a serious effect on the development of depression (23). Moreover, given our findings on bullying, it is important to note that preventing bullying may alter the risk of developing depression in adulthood. In order to reduce the incidence of depression in adulthood, it is necessary to continuously research and contemplate effective measures to prevent bullying.

Bullying trauma in early life had both long-term physical and psychological effects in adulthood (24–26). In addition, previous studies suggested that bullying is a serious social problem that increased the risk of suicide as well as depression (27). Although largely modifiable within schools, monitoring and preventing bullying has become increasingly difficult in recent years due to the growing number of children and adolescents using social media (28). Given the results of our study, attention should be paid to the amount of time children spend on the internet and the types of interactions they are having with other social media users in order to prevent cyberbullying. Bullying prevention programmes, in addition to working within schools, should focus on reducing harm caused by cyberbullying.

Although not statistically significant, psychological trauma showed a marginal association with the risk of developing MDD. This result was, in part, consistent with previous findings that psychological abuse was associated with depression (29). Some previous studies suggested that childhood emotional abuse and neglect were better predictors of adult depression than childhood sexual or physical trauma (10, 13). Consistent with the results of previous studies, we found that psychological trauma was more closely associated with depression than sexual or physical trauma. However, we did not find that emotional neglect trauma was associated with depression.

Unlike previous studies, the insignificant consequences of emotional neglect for developing depression were surprising, given that early life trauma is a significant risk factor for depression. However, not everyone who experiences an early life trauma develops depression in adulthood. Different factors, such as resilience, cognitive function, and emotion regulation ability were shown to mediate the development of MDD from early life trauma (30–32). In addition, strategies for coping with trauma varied from culture to culture, and the symptoms after trauma also changed accordingly (17). In contrast to the familial cultures of the West, Korea had a traditional family-oriented society, which promotes interdependence and cooperation that suppresses the expression of personal feelings and inner mental symptoms (17). Considering that emotional neglect mainly deals with trauma within the family, the participants who grew up in a family-oriented society in Korea may not be sensitive to emotional neglect. Based on these cultural factors in Korea, early life trauma that was perceived as emotional neglect in other countries may not be perceived as emotional neglect by Koreans. Therefore, when evaluating emotional neglect with a simple question, it would have been difficult to reflect Korean familial culture.

In this study, we did not find an association between the development of MDD and sexual trauma. In the results for the frequency of each early life trauma, sexual trauma was reported to be relatively lower than other types of early life trauma, which may be the cause of the low association. Previous studies found that sexual trauma severely affected the victims, both psychologically and physically (33). In addition, previous studies found that sexual trauma affected not only depression but also many other psychopathologies (34, 35). Despite the severe impact of sexual trauma, victims of sexual trauma were less likely to open up to the public than those who experienced other types of trauma (36, 37). Several factors, such as limited support and the perceived negative consequences and feelings of self-blame, prevented disclosure of sexual trauma in early life (38). Also, Korean culture is based on Confucianism, which sees sexuality as taboo and forbid discussion about sex, making it more difficult for people to openly deal with sexual topics (39). Given this, the frequency and consequences of sexual trauma may have been underreported in this study.

The risk of developing MDD was increased as the types of reported early life traumas increased. Individuals exposed to a single form of early life trauma had a high probability of being exposed to other forms of early life trauma (40). Increased early life trauma was associated with more complex adult psychopathology (41). In a South African study, cumulative trauma exposure increased depressive symptoms, and people who experienced multiple traumas were more likely to experience severe symptoms of depression than those who experienced a single event (42). As the frequency of early life trauma increased, the level of depressive symptoms also increased in a dose-response relationship (43).

In participants with MDD, depressed mood showed a marginal association with reported early life trauma. Previous study found that early life trauma was associated with higher levels of somatization in adulthood (44). In addition, alexithymia, which means difficulty in identifying and describing feelings and modulating states of emotional arousal, is associated with somatization (45, 46). People who have experienced early life trauma tend to have lower mentalization, the ability to perceive and interpret human behaviors in terms of intentional mental states (47). Considering the results of these previous studies, depressed patients who have experienced early life trauma may find it difficult to actively express their depressed mood.

Our study has limitations. First, data were obtained from retrospective reports based on participant memories. Thus, biases in memory, such as recall bias, may have affected the accuracy of the data. Depressed individuals could be more likely to remember negative events from their childhood, known as mood-congruent recall (48). Second, since this study was a cross-sectional design, a causal relationship between variables could not be confirmed. Third, early life trauma was evaluated using only simple self-reported questionnaire that were not based on standardized detailed tools. Thus, the intensity and subjective meaning of trauma that can affect result of study could not be identified. Fourth, after Bonferroni correction, some results were not statistically significant and showed a only marginal association. In the results for risk of developing MDD for specific types of reported early life trauma, psychological trauma showed a marginal association. In addition, in the results for comparison of the differential symptoms of MDD according to the presence or absence of early life trauma, depressed mood was marginally associated with reported early life trauma. Fifth, interviewers were educated through mock interview, group discussion and feedback, but inter-rater reliability could not be presented as a statistical value.

Despite these limitations, this study has a strength in that it used a nationwide community sample. In the process of selecting the subjects of the survey, samples were evenly extracted nationwide in order to derive nationally representative results. Results from the Korean representative samples were compared with previous studies, and difference due to cultural factors in Korea was discussed. In addition, strength of this study is that, unlike previous studies, the early life trauma was evaluated separately by type. Through this, each type of early life trauma was comparatively analyzed.

It is important to pay attention to early life trauma to prevent depression. A previous study suggested that social support may moderate the association between early life trauma and poor mental health including depression (6). Considering the results of this study on the relationship between depression and early life trauma, clinicians may need to pay more attention to the type and frequency of early life trauma in depressed individuals. Bullying trauma was particularly related to depression, so clinicians need to pay attention to bullying trauma when interviewing depressed patients. In addition, clinical attention should be paid to the prevention for bullying trauma in school and peer relationships to prevent depression in adulthood. Considering the results of this study that the incidence of depression increased as the type of reported early traumas increased, individuals with early life trauma require early preventive and therapeutic interventions to protect against further trauma. Early intervention for individuals experiencing trauma can help lower the incidence of depression and reduce social costs and burdens.

## Conclusion

We found that the risk of depression varied with the type and frequency of reported early life trauma. Based on these results, early detection and the appropriate management of early life trauma appear important to preventing progression to depression.

## Data Availability Statement

The raw data supporting the conclusions of this article will be made available by the authors, without undue reservation.

## Ethics Statement

The studies involving human participants were reviewed and approved by Seoul National University Hospital. The patients/participants provided their written informed consent to participate in this study.

## Author Contributions

HK contributed to the search for background literature, to writing the original draft of the manuscript, and to reviewing. MP and HP participated in the study design and directed acquisition of the data. MF, DM, and HJJ conceptualized the study and revised the manuscript.

## Funding

This research was funded by the Korean Ministry of Health and Welfare. This study was supported by a grant from the Korean Mental Health R&D Project, funded by the Ministry of Health & Welfare, Republic of Korea (HL19C0001; PI, HJJ), and by a grant from the Korea Health Technology R&D Project through the Korea Health Industry Development Institute (KHIDI), funded by the Ministry of Health & Welfare, Republic of Korea (HR21C0885). The Korean Ministry of Health & Welfare and the NRF had no further role in the study design; in the collection, analysis, and interpretation of the data; in writing of the report, or in the decision to submit the study for publication.

## Conflict of Interest

The authors declare that the research was conducted in the absence of any commercial or financial relationships that could be construed as a potential conflict of interest.

## Publisher's Note

All claims expressed in this article are solely those of the authors and do not necessarily represent those of their affiliated organizations, or those of the publisher, the editors and the reviewers. Any product that may be evaluated in this article, or claim that may be made by its manufacturer, is not guaranteed or endorsed by the publisher.
